# Therapeutic concentrations of glucagon-like peptide-1 in cerebrospinal fluid following cell-based delivery into the cerebral ventricles of cats

**DOI:** 10.1186/2045-8118-8-18

**Published:** 2011-05-17

**Authors:** Silke Glage, Petra M Klinge, Miles C Miller, Christine Wallrapp, Peter Geigle, Hans J Hedrich, Thomas Brinker

**Affiliations:** 1 Institute for Laboratory Animal Science and Central Animal Facility, Hannover Medical School, Carl-Neuberg-Str. 1, 30625 Hannover, Germany; 2 Neurosurgery Foundation, 55 Claverick Str., Providence, RI 02903, USA; 3 International Neuroscience Institute, Rudolf-Pichlmayrstr. 4, 30625 Hannover, Germany; 4 CellMed AG, Industriestrasse 19, 63755 Alzenau, Germany

## Abstract

**Background:**

Neuropeptides may have considerable potential in the treatment of acute and chronic neurological diseases. Encapsulated genetically engineered cells have been suggested as a means for sustained local delivery of such peptides to the brain. In our experiments, we studied human mesenchymal stem cells which were transfected to produce glucagon-like peptide-1 (GLP-1).

**Methods:**

Cells were packed in a water-permeable mesh bag containing 400 polymeric microcapsules, each containing 3000 cells. The mesh bags were either transplanted into the subdural space, into the brain parenchyma or into the cerebral ventricles of the cat brain. Mesh bags were explanted after two weeks, and cell viability, as well as GLP-1 concentration in the cerebrospinal fluid (CSF), was measured.

**Results:**

Viability of cells did not significantly differ between the three implantation sites. However, CSF concentration of GLP-1 was significantly elevated only after ventricular transplantation with a maximum concentration of 73 pM (binding constant = 70 pM).

**Conclusions:**

This study showed that ventricular cell-based delivery of soluble factors has the capability to achieve concentrations in the CSF which may become pharmacologically active. Despite the controversy about the pharmacokinetic limitations of ventricular drug delivery, there might be a niche in this for encapsulated cell biodelivery of soluble, highly biologically-effective neuropeptides of low molecular weight like GLP-1.

## Background

Neurotrophic peptides may have considerable potential in the treatment of acute and chronic neurological diseases. However, systemic delivery is limited by significant systemic side effects, short plasma half-life or poor blood-brain barrier passage. Therefore, local administration into the central nervous system (CNS) has been proposed [1,2]. However, this drug delivery route, bypassing the blood brain barrier, has been challenged, because substances are rapidly removed from the brain through physiological cerebrospinal fluid (CSF) outflow pathways [3-5].

*Ex vivo *gene therapy using encapsulated cell biodelivery has been suggested as a way to achieve a more sustained local delivery of proteins into the CNS. Bankable, non-autologous cell lines cells can be used, that have been genetically engineered to produce the protein. To prevent host versus graft reaction, encapsulation of those cells with permselective membranes has been introduced, to allow diffusional nutrition and the outward passage of the recombinant protein [6]. Encapsulation has been achieved with different techniques, using either hollow fibers or spherical polymeric microcapsules. Hollow fiber encapsulated cells were investigated in clinical trials after implantation into the lumbar subarachnoid space in chronic pain [7] and ALS patients [8] as well as into the lateral ventricle in Huntington's disease patients [9]. While lumbar implantation resulted in excellent cell viability and significant lumbar CSF levels of the secreted soluble factors [7], ventricular implantation has shown disappointing results with variable numbers of surviving cells along with a markedly low secretory rate [9]. Spherical microcapsules may achieve improved viability rates because their shape provides almost ideal diffusion properties which can improve the nutrition of encapsulated cells [10,11].

Glucagon-like peptide-1 (GLP-1) is a neuroprotective substance which has been shown to protect neurons from amyloid toxicity *in vitro *as well as *in vivo *in Alzheimer's disease models [12,13]. Recently, in a rat model of traumatic brain injury, our group has demonstrated similar neuroprotective properties where GLP-1 was delivered from mesenchymal stem cells (MSC) encapsulated in alginate microspheres. After intraventricular implantation of the cells, GLP-1 concentration in the CSF was increased up to 17 pM, while no increased GLP-1 levels were found after transplantation of MSC cell capsules without GLP-1 secretion [14]. Using the same cell capsules in a double transgenic mouse model of Alzheimer's disease, we found a significant anti-inflammatory effect with GLP-1 engineered MSCs, but not with MSC without GLP-1 secretion [15]. To provide further evidence for encapsulated cell biodelivery of neurotrophic substances for clinical applications, the present experiment investigated the viability and secretory activity of cells as well as the cisterna magna CSF level of GLP-1 after cerebral transplantation of GLP-1 producing MSC in an animal model with a larger brain. Three different implantation sites in the cat brain were compared: intraventricular, subdural and intraparenchymal.

## Methods

### Alginate microcapsules and mesh enclosure

**Figure 1:**
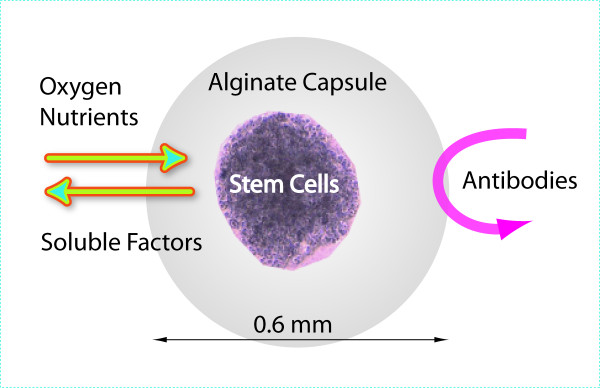
**Illustration of cell encapsulation technology**. Cells are embedded within a polymeric matrix, which is surrounded by an outer cell-free capsule. Smaller molecules diffuse through the capsule, while at the same time, larger proteins and cells are excluded.

A human, bone marrow-derived, mesenchymal stem cell line, manufactured under good manufacturing practice (GMP) conditions, was used in this study. It was immortalized by transduction with the human telomerase reverse transcriptase (hTERT) gene [16]. After transfection with a plasmid vector encoding a GLP-1 fusion gene, the cells produced an 8.7 kDa dimeric GLP-1 molecule. The cells are embedded in a spherical shaped alginate matrix (about 600 μm in diameter). The alginate matrix is generated by cross-linking alginate with barium ions. The alginate itself has no pharmacological effect but provides a mechanical scaffold for the cells and protects them against attacks by the host's immune system. Subsequent to the alginate encapsulation process, the cell capsules were stored in liquid N_2 _until usage. Each capsule contained about 3000 cells. Alginate encapsulation and cell engineering are proprietary manufacturing techniques of CellMed AG, Alzenau, Germany (figure 1).

**Figure 2:**
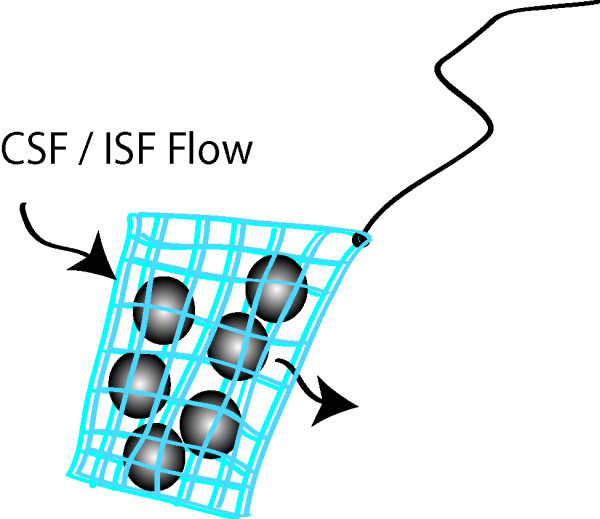
**Diagram of the mesh enclosure**. Several hundreds of cell-containing capsules are packaged in a mesh enclosure that is permeable to fluids. Volumetric flow of cerebrospinal or interstitial fluid through the pores of the mesh supplies internal cells with oxygen and nutrients.

The alginate capsules containing the mesenchymal stem cells were packed within a 1 × 1 cm sized, manually sutured bag using a polypropylene mesh (MB-Mesh 40 Super Light, Medical Biomaterial Products GmbH, Germany). For implantation, 400 ± 100 capsules were thawed and filled into this mesh bag (figure 2). Transplantation within the bag allows for the immobilization of the cell capsules at different intracranial implantation sites. A vicryl thread was attached to the mesh bag to allow retrieval.

### Experimental protocol and surgical techniques (implantation and explantation)

Thirteen cats (European Shorthair, Central Animal Facility, Hannover, Medical School) were used for the experiments. All experimental procedures were performed under general anaesthesia. It was initiated by s.c. injection of a ketaminhydrochloride / xylazine premedication (per kg: 0.1 ml Ketamin Gräub^®^, Dr. E. Gräub AG, Bern, Switzerland; 0,05 ml Rompun^®^, Bayer, Leverkusen, Germany). Subsequently the animals received 2-6ml propofol i.v. (Propofol-Lipuro^®^, B.Braun Melsungen AG, Germany) and, after endotracheal intubation, isoflurane 1.5-2.5% (Forene^®^, Abbot, Baar, Switzerland). All animals received an antibiotic prophylaxis for 10 days (extended-release penicillin und Dihydrostreptomycin^®^, Chassot) as well as an analgetic therapy 3 days post operation (initially 4mg/kg, then 2mg/kg Carprofen s.c., Buprenorphin 0.01mg/kg).

A 0.5 × 1.5 cm frontal craniotomy was performed under sterile conditions below the temporal muscle insertion. A straight dural opening 1.5 cm in length was made. The mesh bag containing the microcapsules was implanted into the subdural (n = 4), the frontal intraparenchymal (n = 5) or the intraventricular (n = 4) space. After a frontal cortisectomy, a subcortical pocket was created for the intraparenchymal placement and a transcortical approach to the frontal horn for the intraventciular implantation of the cell container. Care was given to sufficient hemostasis with bipolar cautery. The dural opening was sealed with absorbable gelatin compressed sponge and the craniectomy was closed with bone wax. The vicryl thread, which was attached to the mesh implant, was incorporated into the bone wax for fixation. The temporal muscle was then reapproximated using 0.3 vicryl sutures and skin was closed with 0.3 monocryl sutures. Antibiotics were given for 10 days post-surgery. The animals were weighed and checked for clinical and neurological symptoms on a daily basis.

Explantation of the mesh was performed after 14 days. The implantation site was explored under general anaesthesia and, once the end of the thread attached to the mesh beg was exposed, the implant was explanted with gentle traction. Blood samples were taken by percutaneous puncture of a forelimb vein, and CSF was collected by percutaneous puncture of the cisterna magna; samples were taken in the subdural and intracerebral implantation group only at day 14, and additionally in the ventricular implantation group at days 0 and 7. After centrifugation, a dipeptidyl peptidase IV inhibitor was added (#DPP4, Biotrend), and the samples were frozen at -80°C until the active GLP-1 was measured by ELISA (Linco, EMD Millipore).

This study was conducted in accordance with the German animal protection law and with the European Communities Council Directive 86/609/EEC for the protection of animals used for experimental purposes. All experiments were approved by the Local Institutional Animal Care and Research Advisory Committee and permitted by the local government (33-42502-06/1133).

### Assessment of stem cell viability during and post-implantation and histological assessment of the implantation site

The explanted cell capsules were recultured and the post-implantation GLP-1 production rate was determined by active GLP-1 ELISA (Linco, EMD Millipore), and compared with the GLP-1 secretion rate prior to implantation. An amount of 10 capsules per explanted containment was transferred into 1ml of culture medium (EMEM, 10% BGS, 2mM L-glutamine) and incubated for 90 min. Capsules were further assessed for viability with SYBR green/propidium iodide staining. After intravital intracardiac perfusion under deep anaesthesia, brains were removed and fixed in 4% paraformaldehyde for histological analysis with hematatoxylin and eosin (H&E) staining.

## Results

### GLP-1 CSF concentrations and stem cell viability

Table 1 summarizes GLP-1 concentrations in CSF and blood plasma, comparing the measures after subdural, intraparenchymal and intraventricular implantation. Cell viability, as obtained by the GLP-1 production rate of stem cells and by the SYBR Green/propidium iodide staining at day 1 post-explantation, is also shown. Viability of cells did not significantly differ between the three implantation sites. Increased GLP-1 concentrations were only found in the CSF, but not in the blood plasma, and only after intra-ventricular implantation both at 7 and 14 days following implantation.

**Table 1:** CSF and serum concentrations of glucagon-1 and cell viability following implantation of encapsulated genetically-engineered stem cells into three intracerebral sites in cats

Implantation Site	Subdural (n = 4)	Intracerebral (n = 5)	Intraventricular (n = 4)
CSF GLP-1 concentration [pM]	14d: 2.3/2.5/<2/2.2	14 d: <2/<2/<2/<2/<2	Day zero: <2/<2/<2/<2 7d: NA**/63/6/73 14d: 47/44/8/<2

Serum GLP-1 concentration [pM]	NA	NA	Day zero: <2.5(all) 7d: <2.5(all) 14 d: <2.5(all)

GLP-1 secretion prior to implantation (fmol/h/capsule)	3.6 (all)	4.6 (all)	4.6 (all)

GLP-1 secretion post implantation (fmol/h/capsule)	NA*/ NA* / 0.6 / 3.0	1.6/ 1.2 / 1.6 / 1.7/ 3.5	0.9/ 1.6/ 1.5 / 1.9

Viability of cells / capsule (SYBRGreen/ propidium iodide)	NA*/NA*/< 10%/40%	53%/58%/20%/83%/70%	41%/52%/51%/53%

NA: Examination not possible (* two bags were histologically investigated after intravital fixation, **CSF puncture not successful). 2.5 pM was the lowest limit of the analysis method.

### Histological findings at the implantation site

**Figure 3:**
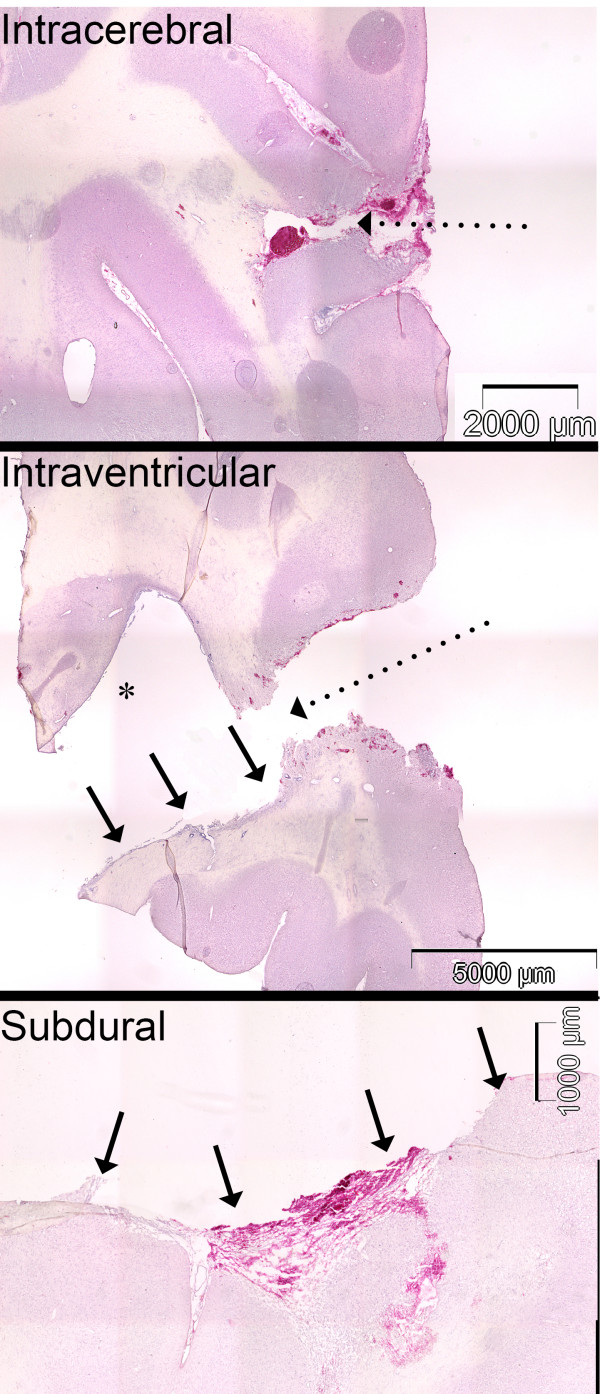
**Coronal cross-sections through the implantation site (H&E stains)**. Minor fresh haemorrhages, as typical after microsurgery, can be seen at the implantation site (arrows) following the explantation procedure. No major gliotic, inflammatory or necrotic areas are visible. Dashed arrows: surgical approach; * = lateral ventricle

Gross macroscopic observation of the explanted brains did not show any significant haemorrhages, midline shifts or signs of infection at the surgical sites. None of the animals exhibited acute occlusive hydrocephalus. Figure 3 shows representative H&E stains for all three implantation sites. H&E staining did not reveal a major local glial or inflammatory tissue response which could have ensheathed the implants. Also, while showing some minor fresh hemorrhages as a result of the surgical retrieval of the mesh bag, the H&E staining ruled out major local tissue injury.

## Discussion

The present study investigates the CSF concentrations of the GLP-1 peptide after implantation of GLP-1 producing encapsulated MSC into the subdural, intraparenchymal and intraventricular space of the cat brain. While viability of cells retrieved after two weeks of implantation did not differ between the implantation sites, the levels of GLP-1 in the CSF were increased after ventricular, but not after subdural or intraparenchymal transplantation. After ventricular implantation, the measured CSF concentrations of GLP-1 reached the level of the binding constant of the GLP-1 receptor [17].

### GLP-1

GLP-1 is an endogenous insulin-stimulating peptide secreted from the gastrointestinal tract in response to food intake [18]. GLP-1 receptors are also expressed throughout the mammalian brain [19]. Stimulation of these receptors is associated with neuroprotective and neurotrophic activity [12,13]. GLP-1 also improves learning and memory, which was shown in GLP-1 receptor-deficient mice [20]. Because the plasma half-life of GLP-1 averages only one to two min, the substance must be locally delivered to the brain despite its good blood brain barrier permeability [21].

We show here that encapsulated cell biodelivery can achieve potentially therapeutic CSF concentration of GLP-1. Our experimental design did not include control groups i.e. capsules with mesenchymal cells but without GLP-1 secretion, because previous experiments in rodents have already shown that only GLP-1 engineered cells, but not native MSCs, achieved increased CSF levels of the peptide [14]. Furthermore, ELISA measurements in the double transgenic mouse model of Alzheimer's disease revealed increased brain tissue concentrations of GLP-1 only after transplantation of GLP-1-secreting capsules. Interestingly, in the transgenic mice a baseline level of GLP-1 was found in untreated control animals as well as after transplantation of MSC without GLP-1 secretion [15]. That increased CSF levels could have resulted from the surgical implantation procedure and the related brain lesion in these experiments, appears to be unlikely as in the traumatic brain injury model we found no increased CSF level of GLP-1 as a result of the brain damage alone [14].

### Viability and secretory activity of retrieved cell capsules

*In vitro *experiments have shown that CSF can serve as a carrier for cellular nutrients [22]. Accordingly, studies in rodents have shown preserved viability and secretory activity after cerebral long-term implantation of micro-encapsulated cells [10,23]. In our cat experiments, we found viability and recorded secretory rates in the retrieved cells, which were as high as those after cerebral transplantation in rodents [14]. The present results indicate that a volumetric flow rate of cerebrospinal or interstitial fluid through the mesh pores exists and that it is sufficient for *in vivo *nutrient and oxygen supply to the encapsulated cells inside the mesh bag. Overall, the highest viability and secretory rates were observed with intraparenchymal transplantation, while after subdural placement they were fairly low. Our present results correspond well to our previous findings in rodents, showing that the secretory activity decreases with the implantation time [14]; future experiments must clarify how long the secretory activity lasts.

### Implantation site

The CSF concentration of GLP-1 was increased after intraventricular, but not after subdural or intraparenchymal implantation. Histological as well as gross macroscopic examination of the brains and the different implantation sites do not suggest that this is secondary to compartmentalization of the implant from local hemorrhage, glial scarring or significant inflammation. Instead, the present findings provide evidence suggesting that in order to obtain efficient CSF concentration of its products, a cell implant needs sufficient and large exposure to the CSF, and one could argue that this would be obtained after intraventricular implantation.

The histological examination of the cat brains revealed a relative large area of brain tissue damage as the result of the implantation of the mesh bag. A clinical translation of this technique would not result in similar lesions, just because of the much larger anatomical dimensions of the human brain. In fact, in the human brain minimal invasive surgical procedures, i.e. endoscopic surgery could be used for the intraventricular implantation of the cell capsules.

### Intraventricular biodelivery with micro-encapsulation techniques

While the intracerebroventricular administration of trophic factors has influenced the pathology of neurodegenerative disorders [24,2], the rapid clearance of CSF into the venous circulation has been recognized as a substantial limitation to the pharmakokinetics of this drug delivery route [4,25]. The only reported clinical study investigating intraventricular, hollow fiber encapsulated cell biodelivery revealed only minimally increased CSF concentration of the delivered factor [9]. The present experiments in a large animal model, however, demonstrate that CSF concentrations up to a therapeutic level can be achieved by ventricular transplantation of cells secreting a soluble factor. This discrepancy can be explained by the different encapsulation technique, i.e. hollow fiber encapsulation *vs*. micro-encapsulation. Micro-encapsulation, as used in our study, allows for the transplantation of a significantly higher number of cells, i.e. millions compared to only hundreds of thousands in the hollow fiber encapsulation [26-28]. Thus, the higher release rates that can be achieved with the micro-encapsulation technique, compensate for the rapid CSF clearance, and thereby help the build up of pharmacologically active CSF factor concentrations. The viability and secretory capacity of our cells at the time of explantation further suggests that the sustained cell-based production of GLP-1 provides steady-state conditions despite a rapid CSF clearance. Having achieved active CSF levels, substances may diffuse into the brain interstitial fluid through the ependymal layer bordering the ventricles. Substances may also be rapidly moved via CSF flow tracks into the hemispheric subarachnoid space, and from there into the brain tissue along the perivascular spaces by means of a rapid convective transport mechanism [29]. These suggestions are supported by the finding that in primates, neurotrophic growth factor can be widely distributed throughout the brain to areas remote from the ventricular infusion site [30]. As encapsulated cell biodelivery has achieved active CSF levels of a soluble factor, this can be considered essential for any pharmacological activity. Nevertheless, because of complex relationships between concentrations in CSF and other brain compartments, CSF concentrations may be difficult to interpret and may have limited value [31]. However, considering our findings in the rodent experiments which showed therapeutic GLP-1 effects after ventricular encapsulated cell biodelivery, GLP-1 might be a prototype for a substance qualified for cell-based delivery into the CSF for the following reasons:

- Its small molecular weight facilitates diffusional and convective distribution through the brain tissue.

- Pharmacological activity is already achieved at considerably low drug concentrations in the picomolar range.

- A sustained delivery may be achieved with encapsulated cell biodelivery.

Despite the controversy about ventricular drug delivery and the discussed pharmacokinetic limitations, there might be a niche in this for encapsulated cell biodelivery of soluble, highly biologically-effective neuropeptides of low molecular weight like GLP-1.

The possibility to explant stem cell transplants from the brain could be an important safety aspect in a clinical application. While retrieval of hollow fibers has been described [1,6], there is currently no non-invasive technique available for the explantation of polymeric microcapsules. Our report is the first to describe a practical technique for the retrieval of microcapsules. We assume that volumetric flow of cerebrospinal or interstitial fluid through the water-permeable mesh permits the diffusional nutrient and oxygen supply for the enclosed cell-containing capsules. Nonetheless, we are aware that the mesh may hamper the cell viability, particularly if the mesh pores are obstructed with invading cells and fibrin and blood clot layers, a finding well known as a cause for ventricular catheter obstruction. This partial clogging of the mesh pores may therefore be responsible for the observed inter-individual variability of viability and GLP-1 secretion rate seen in these experiments.

## Conclusion

This study has shown that cell-based delivery of soluble factors into the cerebral ventricles can achieve pharmacologically active concentrations in the CSF. This finding provides the rationale to pursue the objectives with ventricular drug delivery, particularly to look for possible therapeutic effects of encapsulated cell biodelivery in large animal models of neurological diseases.

## Competing interests

C.W. and P.G. are employed by CellMedAG and T.B. is consultant to CellMed AG, which is the company manufacturing the encapsulated cells.

## Authors' contributions

SG, TB and HJH performed and contributed to the animal experiments; SG, TB, MM and PK contributed to conception, data analysis and preparation of the manuscript, and CW and PG developed and manufactured the cell capsules.

All authors have read and approved the final version of the manuscript.
